# Supplementary material for: Integrating the Idylla™ System Alongside a Real-Time Polymerase Chain Reaction and Next-Generation Sequencing for Investigating Gene Fusions in Pleural Effusions from Non-Small-Cell Lung Cancer Patients: A Pilot Study
Source: Int J Mol Sci. 2024 Jul 11;25(14):7594. doi: 10.3390/ijms25147594 (PMC11277451; doi:10.3390/ijms25147594)
Supplement: Supplementary file 1 [file ijms-25-07594-s001.zip › ijms-3042983-supplementary.pdf]

Supplementary data (S1)

Horizon ALK-RET-ROS1 Fusion FFPE RNA Reference Standard curve

HD784 Horizon is a Reference Standard for ALK-RET-ROS1 Fusion FFPE RNA

1 10 µm FFPE section of HD784 was extracted using the Promega Maxwell® automatic system (AS1360 FFPE RNA Kit) in accordance with manufacturer procedures.

From 1 section we obtained 17,9 ng/ul in 40 ul of mutated RNA.

The extracted RNA was serially diluted (from dilution A to dilution E) with wild type RNA as indicated in the table. 10 ng of DNA necessary for the internal controls of the Idylla™ cartridge was also added to each point of the dilution. 200µl of each dilution combined with 50 µl of RNA Later solution were loaded onto the Idylla ™ cartridge.

Table. Standard curve preparation with HORIZON (HD784)

Dilution	RNA Mut.	RNA WT	DNA control
A	72ng	72ng	10ng
B	36	108	10ng
C	18	126	10ng
D	9	135	10ng
E	4,5	139,5	10ng

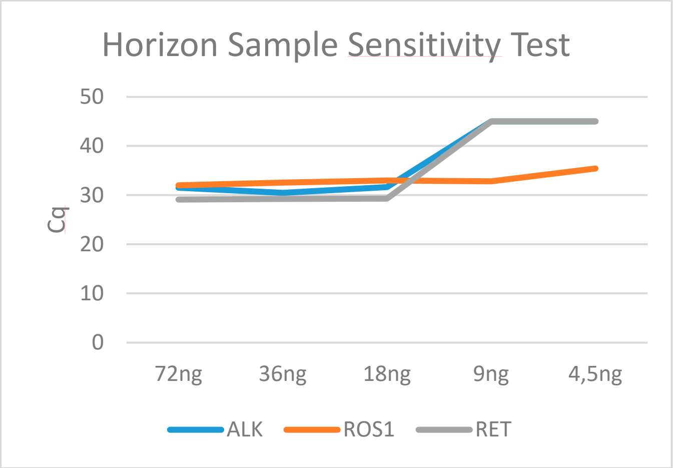

The Horizon standard curve showed that the Idylla ™ system was able to identify fusions by loading at least 15 ng of RNA
